# Integrating hepatitis and HIV point-of-care testing into mandatory migrant tuberculosis screening in the Netherlands: A feasibility and acceptability study

**DOI:** 10.1016/j.puhip.2025.100671

**Published:** 2025-10-15

**Authors:** Chrissy P.B. Moonen, Elfi E.H.G. Brouwers, Casper D.J. den Heijer, Nicole H.T.M. Dukers-Muijrers, Jill Buursma, Inge H.M. van Loo, Christian J.P.A. Hoebe

**Affiliations:** a Department of Sexual Health, Infectious Diseases and Environmental Health, Living Lab Public Health Mosa, South Limburg Public Health Service, Heerlen, the Netherlands; b Department of Social Medicine, Care and Public Health Research Institute (CAPHRI), Maastricht University, Maastricht, the Netherlands; c Department of Health Promotion, Care and Public Health Research Institute (CAPHRI), Maastricht University, Maastricht, the Netherlands; d Department of Medical Microbiology, Infectious Diseases and Infection Prevention, Care and Public Health Research Institute (CAPHRI), Maastricht University Medical Center (MUMC+), Maastricht, the Netherlands

**Keywords:** Point-of-care testing, Hepatitis B, Hepatitis C, HIV, Tuberculosis, Migrants

## Abstract

**Objectives:**

In the Netherlands, tuberculosis (TB) screening is mandatory for migrants from high incidence countries (>200 cases/100,000). These regions often have elevated rates of hepatitis B (HBV), hepatitis C (HCV) and HIV. The INTEGREAT-study assessed the feasibility and acceptability of integrating point-of-care testing (POCT) for these infections into existing TB screening, in line with UN Sustainable Development Goals.

**Study design:**

A mixed-methods study design was used.

**Methods:**

This study offered POCT using finger-prick blood and a brief medical history questionnaire during the public health TB screening. Quantitative data were analysed descriptively. Qualitative data from four focus groups with healthcare workers evaluating implementation were deductively and inductively coded.

**Results:**

Of 293 eligible individuals, 231 (78.8 %) participated. Participants (51.1 % male, mean age 31.9 years) originated from India (27.7 %), South Africa (24.7 %), Pakistan (7.4 %), and other countries (40.2 %). Three participants (1.3 %) tested positive for HIV or HBV and were referred to care if appropriate. Most participants (90 %) preferred finger-prick POCT over venipuncture. Focus groups highlighted the importance of a clear protocol and skilled staff for successful implementation.

**Conclusions:**

This study demonstrated the feasibility and acceptability of integrating POCT into TB screening, although the number of infections was low. Careful follow-up remains essential to ensure effective care.

## 1 Introduction

Communicable infectious diseases such as AIDS, tuberculosis (TB), and hepatitis continue to cause preventable suffering, especially among vulnerable populations [1]. To eradicate these diseases by 2030, the United Nations established Sustainable Development Goals (SDGs) [2]. Progress has stalled in many countries due to inequities in care and the impact of the COVID-19 pandemic. Achieving these targets will require integrated services, increased funding, improved testing access, and targeted support for key populations.

In response to the global elimination appeal, the Dutch National Institute for Public Health and the Environment (RIVM) formulated the National Hepatitis Plan and the National Action Plan on Sexual Transmitted Infections (STI), HIV and Sexual Health [3,4]. These plans aim to further decrease the disease burden and mortality of hepatitis and HIV by identifying bottlenecks and suggesting improvements. Specifically, they emphasise the need for increased accessibility to testing and treatment for hepatitis B virus (HBV), hepatitis C virus (HCV), and HIV among underserved, vulnerable groups and priority populations, including people born in endemic countries (hereafter referred to as ‘migrants’ for consistency) [3,4].

In 2023, 710 TB cases were reported in the Netherlands, with 82 % occurring in migrants. To mitigate the TB transmission risk, the Dutch Foreigners Act mandates TB screening for migrants from high-prevalence countries (>50 cases per 100,000) intending to stay for more than three months, conducted by Public Health Services (PHS) [5]. The mandatory TB screening may also serve as an opportunity to identify blood-borne viruses and other health conditions with public health significance. Compared to the native Dutch population, migrants are approximately 80 % and 60 % more likely to be carriers of chronic HBV and HCV, respectively [6]. In 2018, 104 acute HBV infections were reported in the Netherlands, with 90 % occurring among migrants, while 62 acute HCV cases were identified [7]. In 2022 and 2023, 431 and 424 new HIV cases were reported, respectively, with more than half occurring among migrants [8].

The often asymptomatic nature of HBV, HCV, and HIV highlights the need for early detection through testing, which not only prevents severe health outcomes but also reduces transmission at population level [9]. Additionally, integrated testing for multiple infections improves healthcare efficiency and enhances access to diagnosis and treatment [10].

The World Health Organization (WHO) recommends triple testing for HBV, HCV and HIV, a strategy supported by a recent review involving over 14 million individuals [11,12].

Point-of-care testing (POCT) is an upcoming tool for screening that enables for rapid detection of viral infections near the (asymptomatic) patient [13]. Rapid results enable timely interventions, possibly improving patient outcomes and limiting infection spread. POCT also reduces patient wait times and the likelihood of loss to follow-up. For healthcare providers, it has the potential to enhance resource utilisation efficiency and can be effectively deployed across various settings, including those with limited resources [13].

In the Netherlands, many initiatives aimed at testing migrants for HBV, HCV, or HIV are short-term and project-based [14,15]. Besides, migrants may experience barriers to accessing care, such as language difficulties or cultural differences [4,16]. To explore a potentially sustainable method for triple testing among underserved groups, the INTEGREAT-study implemented an opportunistic POCT offer during an existing healthcare encounter with migrants during their TB screening consultation. Here we report on the evaluation of the integration of POCT using finger prick blood for HBV, HCV and HIV into the mandatory TB screening process among migrants. The study aimed to assess the feasibility and acceptability of triple POCT within existing healthcare settings and to enhance access to testing and appropriate care for underserved migrant populations through an opportunistic, integrated testing approach.

## 2 Methods

### 2.1 Study design and setting

In this cross-sectional mixed-methods study, POCT using finger prick blood detecting HBV, HCV, and HIV and a short medical history questionnaire were offered to migrants during TB screening by X-thorax at the PHS. This quantitative assessment was combined with a qualitative assessment of the implementation process by four focus groups with executive staff. The implementation process was adjusted during the study where necessary. We report on these adjustments, and experienced facilitators and barriers during the implementation.

### 2.2 Study population

#### 2.2.1 Inclusion process

Migrants aged 16 years and over, born in countries with high prevalence rates of HBV, HCV, and HIV, and scheduled for routine TB screening were eligible to participate in the INTEGREAT-study after providing written informed consent. Eligibility was based on the prevalence of TB, HBV, HCV, and HIV in their countries of birth using the most recent country list for Dutch TB screening from the RIVM [17]. The selection process involved several steps: initially, asylum seekers were excluded due to ethical considerations, namely the lack of guaranteed continuity of care in case of infection due to their high mobility and potential institutional barriers such as denial of care [18,19]. A ‘country list’ was made for eligible migrants from which countries were removed of which no individuals had been screened in 2022. For the remaining countries, HBV and HCV prevalence data were added based on information from the Centers for Disease Control and Prevention [20,21]. The risk of STI/HIV was assessed using the RIVM's list of STI/HIV endemic areas [22]. Finally, countries with low HBV prevalence (below 2.0 %) as well as low HCV prevalence (below 1.0 %) were excluded. Countries were included in the screening list if the prevalence of at least two of the specified viruses was classified as intermediate or higher. This resulted in the following included birth ‘country list’: Afghanistan, Angola, Bangladesh, Botswana, Burundi, Cameroon, Eritrea, Ethiopia, Gambia, Ghana, India, Indonesia, Ivory Coast, Kyrgyzstan, Mongolia, Myanmar (Burma), Nigeria, Pakistan, Philippines, Somalia, South-Africa, Tanzania, Thailand, Uganda, Vietnam, Zambia, and Zimbabwe.

The migrant's eligibility for the study was verified by the PHS employee when they contacted the call centre to schedule a TB screening consultation. Eligible individuals were assigned a participant number to ensure confidentiality and a note was added to the medical record to ensure offering the tests to eligible individuals during TB screening. All eligible migrants received a bilingual (English and Dutch) brochure written in simple (B1 level) language, providing information about the study, the infectious diseases, the POCT, and treatment options.

### 2.3 Data collection

#### 2.3.1 Diagnostic outcomes

Participants were tested using POCT for hepatitis B surface antigen (HBsAg), anti-HCV, and anti-HIV markers. These POCTs were offered during 15 testing days between January 30, 2024 and August 13, 2024. The reasons for not participating were documented. Capillary blood samples from participants were obtained by skilled public health nurses following their TB screening appointment. All nurses involved in the study had extensive experience serving a diverse migrant population and received specialised training on the POCT procedures and protocols to ensure consistency and accuracy in data collection. The following POCTs were used according to the manufacturer's protocols: the DETERMINE™ HBsAg 2 by Abbott (Chicago, United States), the HCV Rapid Antibody Tests by OraSure (Bethlehem, United States), and the DETERMINE™ HIV EARLY DETECT by Abbott (Chicago, United States). These tests meet the European Union (EU) and WHO requirements for analytical sensitivity and specificity [[23], [24], [25]]. After 20 min, the nurse read and recorded the test results.

After the POCTs were performed, participants completed a one-page questionnaire together with the nurse, which was available in both English and Dutch. The questionnaire collected information on basic demographics, test and infection history, treatment and HBV vaccination status, reason for participation, preferred sampling method, and their assessment of the POCT experience, including a rating on a scale from 1 (very unsatisfied) to 10 (very satisfied), and an elaboration on their rating (“Why did you give this rating?”).

In line with regular TB screening practices, results were communicated using the “no news is good news” principle. Participants with a positive POCT result who were not already receiving treatment were contacted by phone. Subsequently, they received a letter instructing them to obtain a venous blood sample at the nearest hospital for confirmation. Confirmation tests were performed on venous blood (HBsAg, (Elecsys HBsAg, Roche, Switzerland), anti-HCV (Elecsys anti-HCV, Roche Switzerland) and HIV Ag/Ab (Elcsys HIV combi PT, Roche, Switzerland). Subsequently, regular care commenced, including contact with an infectious disease medical specialist, source and contact tracing where indicated and linkage to appropriate care.

#### 2.3.2 Process evaluations

The process was evaluated through four focus groups held at four key points: one just after the start, two during, and one immediately after the completion of the study. These focus groups were conducted at the PHS with the set project group of eight staff members, which included researchers, research nurses, infectious disease doctors and TB physician assistants. Depending on availability, four to six staff members participated in each focus group, in which at least one of the different roles was represented. The discussions, with the lead researcher as moderator, focused on the current state of the study, experienced barriers and facilitating factors in implementation, and key learning points. Audio recordings of the discussions were made and transcribed verbatim.

### 2.4 Data analysis

Quantitative data analysis was conducted using the Statistical Package for the Social Sciences (SPSS, version 27.0, IBM, Armonk, USA) for Windows. Upon study completion, the paper questionnaires and test results were entered deidentified and coded into the online survey tool Crowdtech. Descriptive data analysis was performed, and participants were compared with non-participants and no-shows on sex, age group, and country of birth using the Pearson Chi-Square test. When more than 20 % of the cells had an expected count of less than 5, the Likelihood Ratio Chi-Square Test was used. Statistical significance was considered at a p-value of 0.05 or less. We divided the sample into two age categories: 16–29 years and 30 years and older. Based on the median of 30 years, this division allowed for the analysis of age-related differences between the participants and non-respondents.

Thematic analysis of the focus groups was conducted using ATLAS.ti (version 22) through both deductive and inductive coding. CM was the first coder, and JB was the second coder. The first transcript was coded independently by CM and JB to ensure consistency and alignment in the coding process. Subsequent transcripts were primarily coded by CM and subsequently counter-coded by JB. Any discrepancies in interpretation were discussed and resolved through consensus. Following the coding phase, the identified factors were systematically organised into a model guided by the social-ecological model [26]. This model was further reviewed and refined in collaboration with the co-authors. The significance of each concept was determined based on its frequency of occurrence across the focus group discussions.

## 3 Results

### 3.1 Reasons for (non) participation

The participants (n = 231) were asked to indicate their reasons for participating in the POCT tests. The most common responses were a desire to know their infection status (52.4 %), wanting to contribute to the research (29.9 %), no specific reason (17.7 %), and being asked by the health professional (5.6 %).

Among non-participants (n = 62), the main reasons for declining the POCT offer were already being tested for these infections (46.8 %), and fear of needles or blood tests (16.1 %). Other commonly mentioned reasons included no time (9.7 %), no benefit of the test (9.7 %), hearing about the research for the first time (4.8 %), language barriers leading to misunderstanding (3.2 %), and need for family consultation first (3.2 %).

### 3.2 Study population and characteristics

**Fig. 1:**
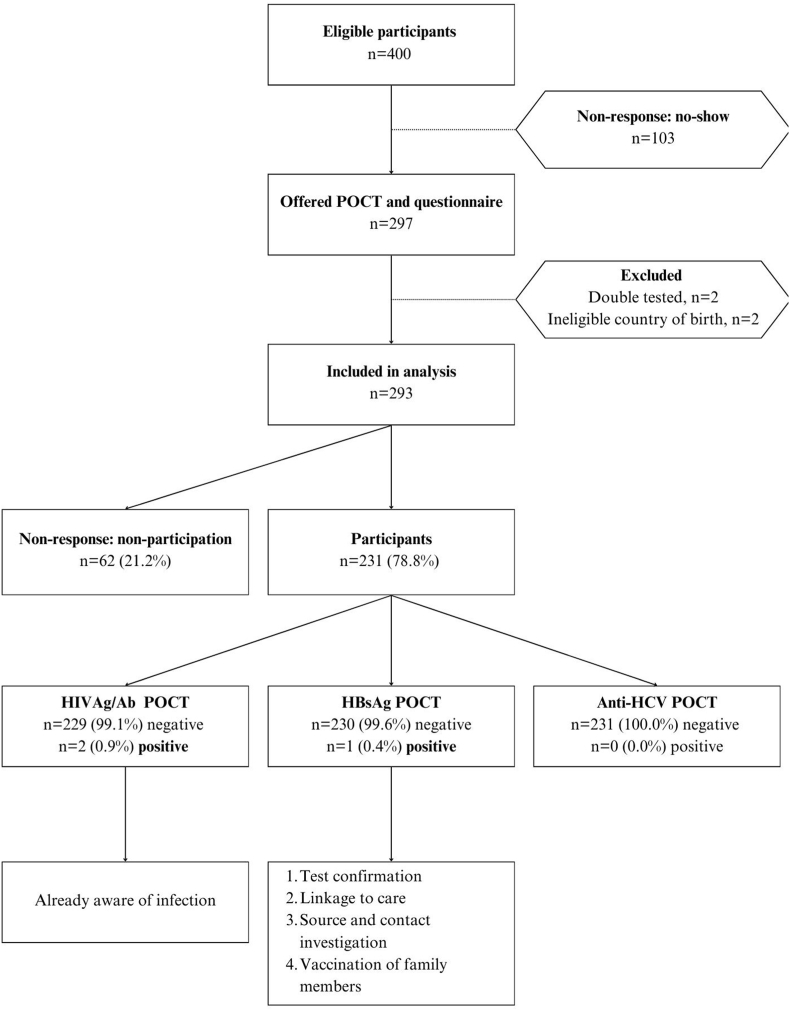
Flowchart of participation and test results.

Of the final study population meeting the inclusion criteria (n = 293), 231 participants (78.8 %) were successfully tested (Fig. 1). The mean age of the participants was 31.9 years, and approximately half were male (51.1 %) (Table 1). Migrants from twenty different countries were tested, the majority originating from India (27.7 %), South Africa (24.7 %), and Pakistan (7.4 %). The participants did not differ from the non-responders regarding sex (*p* = 0.530) and age (*p* = 0.277) (Supplement 1).

**Table 1:** Demographic characteristics of the participants and non-response.

	Participants N (%)	Non-response N (%)
Sex		
Male	118 (51.1)	79 (47.9)
Female	113 (48.9)	86 (52.1)

**Age**		
Under 30	109 (47.2)	87 (52.7)
Over 30	122 (52.8)	78 (47.3)

**Country of birth**		
Afghanistan	4 (1.7)	1 (0.6)
Bangladesh	1 (0.4)	6 (3.6)
Cameroon	2 (0.9)	0 (0.0)
Eritrea	3 (1.3)	5 (3.0)
Ethiopia	4 (1.7)	4 (2.4)
Ghana	3 (1.3)	1 (0.6)
India	64 (27.7)	79 (47.9)
Indonesia	16 (6.9)	15 (9.1)
Kyrgyzstan	1 (0.4)	0 (0.0)
Myanmar (Burma)	4 (1.7)	0 (0.0)
Nigeria	13 (5.6)	13 (7.9)
Pakistan	17 (7.4)	13 (7.9)
Philippines	16 (6.9)	5 (3.0)
Somalia	1 (0.4)	3 (1.8)
South-Africa	57 (24.7)	15 (9.1)
Thailand	13 (5.6)	0 (0.0)
Uganda	2 (0.9)	0 (0.0)
Vietnam	6 (2.6)	1 (0.6)
Zambia	2 (0.9)	2 (1.2)
Zimbabwe	2 (0.9)	2 (1.2)

### 3.3 Testing, infection, and vaccination status

Three individuals had a positive test result, of which two tested positive for HIV Ag/Ab and one for HBsAg. While the individuals were already aware of their infections, and the two with positive HIV tests were already receiving care, the individual with a positive HBsAg test had not yet received treatment. Following confirmation, the individual was linked to care, and source and contact tracing were conducted according to national guidelines. In addition to the individual with a positive test result, another reported a prior HBV infection that had resolved naturally.

Prior to study entry, 71.4 %, 65.4 % and 51.9 % of participants had never been tested for HCV, HBV and HIV, respectively (Table 2). Of the previously tested, around 80 per cent were tested in the country of birth. Approximately half of the tested participants were tested in the last three years. Only 23.8 % indicated with any certainty that they had been HBV vaccinated.

**Table 2:** Previous test-, infection-, and vaccination status.

Characteristics	HBV N (%)	HCV N (%)	HIV N (%)
**Previously tested**	**n**=**231**	**n**=**231**	**n**=**231**
No	151 (65.4)	165 (71.4)	120 (51.9)
Not sure	24 (10.4)	25 (10.8)	14 (6.1)
Yes	56 (24.2)	41 (17.7)	97 (42.0)

**Country of test**	**n**=**45**	**n**=**33**	**n**=**84**
Country of birth	37 (82.2)	26 (78.8)	73 (86.9)
Netherlands	3 (6.7)	2 (6.1)	5 (6.0)
Other country	5 (11.1)	5 (15.2)	6 (7.1)
**Year of test**	**n**=**33**	**n**=**23**	**n**=**71**
Between 2022 and 2024	17 (51.5)	10 (43.5)	37 (52.1)
Between 2019 and 2021	5 (15.2)	6 (26.1)	21 (29.6)
Between 2016 and 2018	6 (18.2)	4 (17.4)	7 (9.9)
2015 or longer ago	5 (15.2)	3 (13.0)	6 (8.5)

**Previous test result**	**n**=**56**	**n**=**41**	**n**=**97**
Negative	53 (94.6)	41 (100.0)	95 (97.9)
Not sure	1 (1.8)	0 (0.0)	0 (0.0)
Positive	2 (3.6)	0 (0.0)	2 (2.1)
**Treated**	**n**=**2**	**n**=**0**	**n**=**2**
No	2 (100.0)	0 (0.0)	0 (0.0)
Yes	0 (0.0)	0 (100.0)	2 (100.0)

**HBV vaccinated**	**n**=**210**	**-**	**-**
Yes	50 (23.8)	**-**	**-**
No	83 (39.5)	**-**	**-**
Not sure	77 (36.7)	**-**	**-**

### 3.4 Experience of POCT

Of the participants (n = 231), 90 per cent indicated to prefer finger prick blood sampling over venipuncture sampling. The remaining participants had no preference (6.5 %) or preferred venipuncture sampling (3.5 %). Participants rated their experience with POCT by finger prick blood sampling on a scale from 1 (very unsatisfied) to 10 (very satisfied), resulting in a mean score of 8.7. (SD = 1.5, range = 4–10, 95 % CI [8.52, 8.91]). When asked to elaborate on their score, the most indicated reason was that ‘the tests were easy’ (24.4 %). As for people that related their score to pain, responses varied: the majority indicated that it ‘‘didn't hurt (that much)’’ (38.7 %), while some described it as ‘‘(a little bit) painful’’ (11.6 %). Additionally, 14.7 % cited the rapid execution of the tests as a reason for their positive evaluation.

### 3.5 Qualitative assessment

**Fig. 2:**
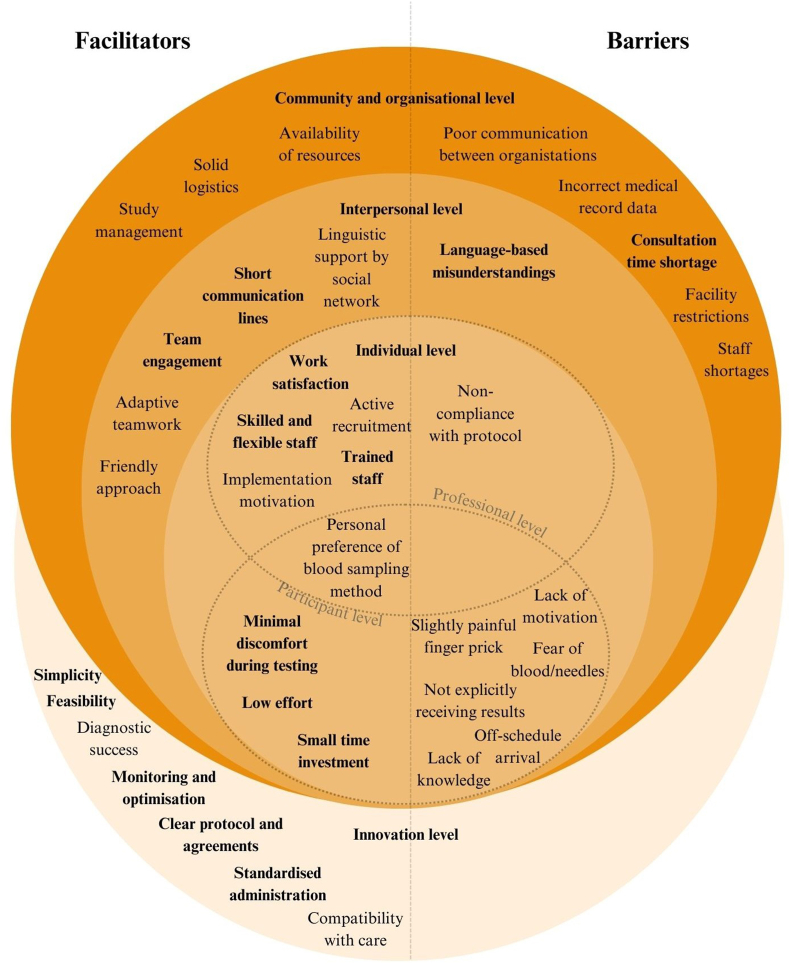
Facilitating factors and barriers in the implementation of the INTEGREAT-study with major themes in bold.

Fig. 2 presents an overview of the facilitators and barriers identified through the study's qualitative assessment, which was the result of the focus groups, with major topics in bold. These factors were categorised according to multiple levels of influence, derived from the socio-ecological model [26]. Notably, most of the identified factors were situated at the individual level.

#### 3.5.1 Innovation level

A jointly developed protocol guided implementation, ensuring alignment with standard care workflows for sustainability and minimal staff burden. Key measures included the deployment of the existing “no news is good news” approach for test results and routes for lab requests and source and contact tracing. Regular evaluations through structural updates and focus groups allowed continuous optimisation. For instance, the medical history questionnaire was administered orally alongside POCT to maintain patient flow, and POCTs were closely monitored due to fluid stream issues. Despite minor challenges, POCT was simple, feasible, and consistently yielded valid results. Standardised administration reduced errors and ensured clear study oversight.

#### 3.5.2 Individual level: professional level and participant level

Healthcare professionals demonstrated skill and flexibility, supported by their experience with the target population. Comprehensive training and pre-implementation preparation, including POCT practice, facilitated smooth execution. Enthusiastic staff recruitment likely contributed to high participation, with participants citing being requested by the staff as their motivation to join. Staff appreciated expanding their roles and found the study rewarding, enhancing implementation success. However, occasional protocol non-compliance led to premature exclusions, though this was swiftly corrected through the structural evaluations.

Both professionals and participants preferred finger-prick testing over venous sampling due to its practicality and ease of use, even for those with difficult-to-access veins.

Participants generally found finger-prick testing minimally painful and the process effortless. However, key barriers included: 1. Fear of needles or blood, causing discomfort, 2. Limited knowledge about HBV and HCV, including their own infection and vaccination status, 3. Limited awareness of the POCT offer prior to the appointment due to low engagement with the informational leaflet, which participants often reported not having read when asked verbally (reason unknown), and 4. Irregular attendance at TB appointments, causing unpredictability in participant numbers.

#### 3.5.3 Interpersonal level

Language barriers reduced mutual understanding, though accompanying family or acquaintances often provided linguistic support. Participants perceived the staff as kind and approachable. Among professionals, teamwork was effective, with adaptability to colleagues’ working styles. Short communication lines via email, phone, and face-to-face interactions enhanced cooperation and transparency.

#### 3.5.4 Community and organisational level

Logistics and management were effective, ensuring a smooth workflow and material availability. However, several barriers emerged: 1. Errors in medical records regarding country of birth affected eligibility assessments, 2. Time constraints during consultations limited participant education, 3. Staff shortages in TB services, exacerbated by maternity leave and holidays, impacted capacity, 4. Delayed communication of lab test results between hospitals and infectious disease units hindered care continuity and 5. Facility limitations, as the PHS lacked its own TB screening site, relying on a regional PHS with restricted space, reducing testing frequency and prolonging the study.

## 4 Discussion

The INTEGREAT-study illustrated the feasibility of integrating POCT for HBV, HCV, and HIV within the mandatory TB screening of migrants. The high participation rate (78.8 %) and predominantly positive experiences of POCT by staff and participants suggest that an integrated testing offer can be a low-barrier approach to expanding infectious disease detection in at-risk populations.

While the POCT intervention was feasible and acceptable, the observed infection rate (1.3 %) was lower than expected based on the prevalence rates in the participants' countries of origin. However, identifying and managing even a single undiagnosed infection remains crucial in the context of a zero-hepatitis and HIV elimination policy. This low observed infection rate may be attributed to several factors. Firstly, the HBsAg POCT utilised in this study detected only active infections, providing no insights into past, resolved infections. Secondly, our study focused exclusively on labour and student migrants, who might be at lower risk for HBV, HCV or HIV compared to asylum seekers [27]. Lastly, the observed low infection rate might be explained by a possible ‘‘Healthy Migrant Effect’‘, which refers to the phenomenon that migrants tend to be healthy and resilient members of their population [28]. This self-selection bias may contribute to a lower prevalence of infectious diseases and other health conditions among migrants than anticipated.

Quantitative and qualitative evaluations revealed key insights for implementation. The POCT was well accepted by both participants and professionals due to the minimal invasiveness of the finger prick and the easy and quick administration of the tests, echoed by a previous study among Syrian migrants [15]. The successful implementation of this study relied heavily on thorough preparation, which included establishing clear protocols, defining precise agreements, and ensuring effective communication among trained and skilled team members. Continuous monitoring and ongoing optimisation only enhanced team engagement and contributed to quality improvement and the standardisation of protocols. Recurring barriers encountered during implementation included limited consultation time and language-based misunderstanding. Given the limited time allocated for each appointment and the unpredictability of migrants arriving outside their scheduled times, utilizing pre-booked interpreters or online translation tools proved impractical. This underscores the need for strategies to address these challenges in future initiatives, such as allocating more time per consultation and linguistic support to facilitate comprehensive information delivery.

Besides the recurring barriers, it should be noted that within the study population, there was a lack of knowledge about hepatitis, including (HBV) vaccination and infection status. Informational brochures were rarely read by participants, indicating that other pre-intervention communication strategies might be more effective. Furthermore, independent of the study, high no-show rates are common within this setting. Besides, analysis of both qualitative and quantitative data revealed that migrants were regularly tested in their home countries, particularly in India, Pakistan, South Africa, and the Philippines. For migrants originating from these regions, the utility of such tests might be limited.

By offering POCT, infectious disease testing becomes more accessible to underserved populations, which might contribute to reducing health inequalities among marginalised groups. The findings indicate that the POCT likely contributed to the high participation rate due to its minimally invasive nature. POCT has also been proven feasible in other Dutch settings, such as community-based HIV testing [29]. Ensuring follow-up care for individuals with positive results remains a key concern. Effective linkage to care is vital to translating testing outcomes into long-term health benefits. Efforts should prioritise improving continuity of care, especially for vulnerable migrant populations [30]. To foster sustainable integration, the implementation was designed to align with existing healthcare workflows. However, a notable delay was observed in communicating confirmation test results from the laboratory to the PHS’ infectious disease unit. Without close follow-up, such delays could significantly delay care initiation and even risk lost to follow-up. Exploring strategies to enhance follow-up and remove participation barriers for specific migrant subgroups is crucial. Finally, conducting an economic analysis could offer valuable insights into the cost-effectiveness of this approach.

### 4.1 Strengths and limitations

Key strength of this study was the absence of selective dropout based on age or sex, ensuring the study population's representativeness. The mixed-method design provided valuable insights beyond the descriptive data, shedding light on factors that both hinder and facilitate the implementation process. Enabling the rapid and effective detection of infections by professionals with minimal training can strengthen public health responses. However, this study has also several limitations. The self-reported medical history is subject to recall bias, and the oral administration of the questionnaire may lead to misunderstandings and missing data, particularly among participants with language barriers. Furthermore, this study had a relatively small sample size and was only applied in a single region, limiting generalisability.

### 4.2 Conclusions

The INTEGREAT-study, in which triple POCT was implemented in the existing TB screening for migrants, proved feasible and acceptable by both participants and staff. This method facilitates infectious disease testing in harder-reached migrant populations, aligning with global HBV, HCV, and HIV elimination goals. Further research, including cost-effectiveness analyses, is needed to assess the broader value of implementation in routine practice.

## Ethical approval

The study protocol, participant information and written informed consent form were approved by the Medical Ethical Committee of Maastricht University Medical Centre in Maastricht, the Netherlands (reference number METC 2023-0315).

## Availability of data and materials

The data of this study contain potentially identifying and sensitive participant information. Due to the General Data Protection Regulation, it is not allowed to distribute or share any personal data that can be traced back (direct or indirect) to an individual. In addition, publicly sharing the data would not be in accordance with participants’ consent obtained for this study. Therefore, data used and/or analysed during the study are available from the head of the data-archiving of the Public Health Service South Limburg on reasonable request. Interested researchers should contact the head of the data-archiving of the Public Health Service South Limburg (Tamara Kleine: tamara.kleine@ggdzl.nl) when they would like to re-use data.

## AI

During the preparation of this work the authors used ChatGPT (OpenAI Inc) version 3.5 in order to improve the correctness of the language and reduce the word count. After using this tool, the authors reviewed and edited the content as needed and take full responsibility for the content of the publication.

## Funding

This work was financially supported by 10.13039/501100001826ZonMw, The 10.13039/501100001718Netherlands Organisation for Health Research and Development [10710032310005, 2023]. The funders had no role in study design, data collection and analysis, decision to publish or preparation of the manuscript.

## Declarations of interest

None.
