# Supplementary material for: Integrating hepatitis and HIV point-of-care testing into mandatory migrant tuberculosis screening in the Netherlands: A feasibility and acceptability study
Source: Public Health Pract (Oxf). 2025 Oct 15;10:100671. doi: 10.1016/j.puhip.2025.100671 (PMC12556238; doi:10.1016/j.puhip.2025.100671)
Supplement: Multimedia component 1 [file mmc1.docx]

**Supplement 1.** Comparison of participants and non-participants on sex and age

|  | **Participants**  **N (%)** | **Non-participants N (%)** | **(Value, df), P-value** |
| --- | --- | --- | --- |
| **Sex** |  |  | (0.395, 1) 0.530 |
| Male | 118 (51.1) | 79 (47.9) |  |
| Female | 113 (48.9) | 86 (52.1) |  |
| **Age** |  |  | (1.182, 1) 0.277 |
| 16-29 | 109 (47.2) | 87 (52.7) |  |
| 30+ | 122 (52.8) | 78 (47.3) |  |
